# The Effect of *Centella asiatica* Methanolic Extract on Expression of IL-1β Proinflammatory Cytokines in Severe Early Childhood Caries

**DOI:** 10.1055/s-0042-1748528

**Published:** 2022-08-09

**Authors:** Muhammad Luthfi, Fathillah Abdul Razak, Devy Putri Kusumawardhani, Ayu Anggraini Broto Nagoro, Naura Fadhila

**Affiliations:** 1 Department of Oral Biology, Faculty of Dental Medicine, Universitas Airlangga, Surabaya, Indonesia; 2 Department of Oral and Craniofacial Sciences, Faculty of Dentistry, University of Malaya, Kuala Lumpur, Malaysia; 3 Department of Oral Biology, Faculty of Dental Medicine, Universitas Airlangga, Surabaya, Indonesia

**Keywords:** *Centella asiatica*, S-ECC, IL-1β, chronic diseases, medicine

## Abstract

**Objective** This article analyzes the role of *C. asiatica* extract in reducing the proinflammatory cytokine interleukin (IL)-1β produced by salivary neutrophils.

**Material and Methods** Selected kindergartens in the Surabaya area provided samples. The sample was initially checked for dental caries by measuring its def-t index, and then the participants who satisfied the requirements for severe caries with a def-t of greater than 6 were chosen. At the time of sampling, all of the individuals were between the ages of 4 and 6. The sampling was performed by researchers and certified persons using well-known methodologies. For 60 minutes before to sampling, respondents were not allowed to eat, drink, chew gum, or brush their teeth. For analysis, the samples were collected and then frozen at −80°C.

**Results** The administration of methanolic extract *C. asiatica* decreased the expression of the proinflammatory cytokine IL-1β on the surface of salivary neutrophils on S-ECC; The administration of *C. asiatica* methanol extract resulted in a decrease in the expression of the proinflammatory cytokine IL-1β on the surface of salivary neutrophils in S-ECC.

**Conclusion** *C. asiatica* extract has the effect of reducing the proinflammatory cytokine IL-1β produced by salivary neutrophils on S-ECC via inhibiting nuclear factor kappa B and mitogen–activated protein kinase signaling pathway activation and suggest that *C. asiatica* is a possible candidate for treating S-ECC.

## Introduction

Dental caries is a multifactorial, chronic condition that begins with a microbiological alteration in a complex biofilm driven by sugar intake, salivary flow, and behavior. 1 The long-term alter of plaque flora against acidogenic and aciduric bacteria, resulting in a low plaque pH, followed by a high intake of sucrose-containing foods and the presence of factors such as oral hygiene, aging, genetic factors, and immune system changes, all of which create conditions in the body. Some acidogenic and aciduric species, such as *Streptococcus mutans* or *Lactobacilli* , thrive in plaque. 2

Severe early childhood caries (S-ECC) is a highly destructive form of dental caries with decay exfoliation filling teeth (def-t) > 6, involving several teeth, including maxillary anterior teeth with signs of smooth surface decay in children under the age of 3. It usually begins soon after the first tooth erupts and progresses rapidly to the cavity stage in as little as 6 to 12 months. 3 Although scientific evidence suggests that treating ECC improves children's and parents' quality of life, there are still over 621 million children worldwide who have dental caries. 4 5

In low-income countries, the prevalence of ECC approaches 85% in financially deprived people. 6 According to the American Academy of Pediatric Dentistry, caries affects 70% of children aged 2 to 5. With a def-t index > 6, Indonesia falls into the S-ECC category, with a prevalence of caries at the age of 5 years of 67.3%. 7 This demonstrates that preventive strategies in the management of dental caries in kids under the age of 6 years have failed. The great incidence of the condition, as well as the individual and communal degrees of the disease, makes ECC prevention and treatment a public health issue. 8

In recent years, the public's image of neutrophils has altered dramatically, with neutrophils now being recognized as a crucial component of the body's first line of defense against microbes. 9 Neutrophils not only assist phagocytes in destroying germs by releasing numerous reactive oxygen species and antimicrobial peptides, but they also play a role in controlling immune response activation. 10 Furthermore, neutrophils have been reported to produce cytokines, chemokines, and growth factors, making them a significant contributor to the generation of proinflammatory cytokines in the infection region. 11

*Centella asiatica* is one of the plants that is widely known as medicinal herb. Asiaticoside, brahmoside, asiatic acid, and brahmic acid (madecassic acid) are pentacyclic triterpenoids found in *Centella* ; other compounds include centellose, centelloside, and madecassoside. The triterpenoid, saponins of *C. asiatica* , showed immunomodulatory effect, 12 while pectin isolated from *C. asiatica* showed immunostimulant activity 13 and methanol extract showed immunomodulatory activity. 14 The ethanolic extract of *C. asiatica* activates the cell-mediated immune system which enhances the phagocytic function of neutrophils. 15 According to those reasons, this study aims to determine the effect of methanolic extracts *C. asiatica* as an immune modulator in increasing the role of salivary neutrophil cells as a component of innate immunity in preventing the occurrence of S-ECC via inhibiting cytokine proinflammatory release.

## Material and Methods

### *Centella Asiatica* Extract Preparation

*C. asiatica* plant was obtained from the plantation of UPT Materia Medica Batu, Malang City, East Java, Indonesia, washed, and then cut into small pieces and dried in a drying oven at 50°C, a constant weight was obtained. The dried *C. asiatica* plant is then ground into a powder. Two grams of okra powder was extracted with 20 mL of 70% methanol solvent at a ratio of 1:10 (w:v) during the maceration period, that is, 24 hours at room temperature and centrifuged at 150 revolutions per minute (rpm). After the maceration period, the soaked powder solvent mixture was filtered using filter paper and then concentrated into 1 mL with a rotary evaporator at a temperature of 40 to 60°C. Then diluted with dimethyl sulfoxide 5% at a temperature of 1:1 (v:v) which produces okra fruit extract with a concentration of 100%, then diluted to 12.5, 25, 50, 100, and 200%. The liquid extract of okra fruit was then stored at –20°C. 16

### Sampling

This research has been approved by the ethics committee of the Faculty of Dental Medicine, Universitas Airlangga, Surabaya, Indonesia (No. 255/KKEPK/FKG/XI/2016) and granted ethical clearance. Selected kindergartens in the Surabaya area provided samples. The sample was initially checked for dental caries by measuring its def-t index, and then the participants who satisfied the requirements for severe caries with a def-t of greater than 6 were chosen. At the time of sampling, all of the individuals were between the ages of 4 and 6. Before collecting samples from the study participants, they were given questionnaires to complete to screen for caries from a socioeconomic aspect, and their parents signed an informed consent form. The sampling was performed by researchers and certified persons using well-known methodologies. For 60 minutes before to sampling, respondents were not allowed to eat, drink, chew gum, or brush their teeth. For analysis, the samples were collected and then frozen at –80°C.

### Isolation of Salivary Neutrophil Using Magnitude Beads and Cd177 Expression with Flow Cytometry

The participant was told to gargle with 10 mL of sterile natrium chloride (NaCl) solution for 30 seconds while gargling but not ingesting, then wait orated in a sterile glass to collect neutrophils in saliva. This procedure was repeated four times. After that, the samples were centrifuged at 450 × *g* for 15 minutes at 40°C. The centrifuged pellet was then mixed with 2 mL of RPMI 1640 medium, and neutrophils were identified using an EasySep Human Neutrophil Enrichment Kit sorting cell. 17

Flow cytometry was used to examine cell suspensions using fluorescence activated by cell FACScan analyzer (Becton Dickinson). Depending on the size and granularity of the neutrophil suspension, each sample of salivary neutrophils was determined to obtain the profile of those neutrophils using forward scatter (FSC) and side scatter (SSC). Positive staining for the neutrophil marker was used to characterize events beyond the degree of fluorescence in cells that had been determined by their characteristic. Neutrophils that contained more than 70% of the isotope and were matched to a staining control were investigated. By subtracting isotope positive staining cells from positive staining antibody cells, the proportion of neutrophil cells was calculated. In the meantime, the percentage of luminous neutrophil cells was measured by gating on both those cells that reacted negatively to the labeled propidium iodide. The morphology of cells labeled positive was then verified by comparing the back gating for FSC to the SSC plot.

### Analysis of Effects of *Centella asiatica* Extract on Interleukin-1β Expression in Neutrophil Cells with Flow Cytometry

According to Vasilyev et al, 18 the flow cytometry procedure used to evaluate the expression of interleukin (IL)-1β on the surface of salivary neutrophils is as follows.

## Immunostaining

At room temperature, saliva suspension was centrifuged for 10 minutes at 1,300 rpm. The cells were washed twice in 1 mL phosphate-buffered saline (PBS) containing 1% bovine serum albumin (BSA). Following that, 25L cell (1 × 10 ^5^ ) and 1L (3 µg) human immunoglobulin G were combined in designated 12 × 75-mm flow tubes and incubated at room temperature for 20 minutes in the dark. Anti-IL-1β antibodies were added in a saturating concentration of 10 µL each. Finally, antibodies against anti-CD177C and anti-human IL-1β were used to immunophenotype salivary neutrophil subpopulations. The cells and antibodies were incubated in the dark for 40 minutes at 4°C. In 1 mL of PBS with 1% BSA, the cells were washed twice to eliminate unbound antibodies. Following that, 100 mL of PBS was added to the tubes, and the contents were examined by flow cytometry without fixation right away.

### Flow Cytometry Analysis

BD-FACS flow cytometer (fitted with three lasers—488, 633, and 405 nm) and FACSDiva software version 6.1.2 were used for flow cytometric analysis (BD Biosciences). Researchers divided the populations to be studied into lymphocytic and monocytic regions based on forwarding and side scattering indices (FSC-A/SSC-A dot plot). Following that, we used markers from the distinct subpopulations to gate CD177 subpopulations (dot plots APC-A/FITC-A and APC-A/PE-Cy7-A). A total of 10,000 occurrences had to be gated. Then, using phycoerythrin/count histograms, we calculated the percent of positive events and mean fluorescence of cells carrying membrane-bound receptors for each of these subpopulations, using an interval gate on the control histogram gained with samples incubated in the absence of anti-human IL antibodies. 19

## Result

See Figs. 1 2 3 .

**Fig. 1:**
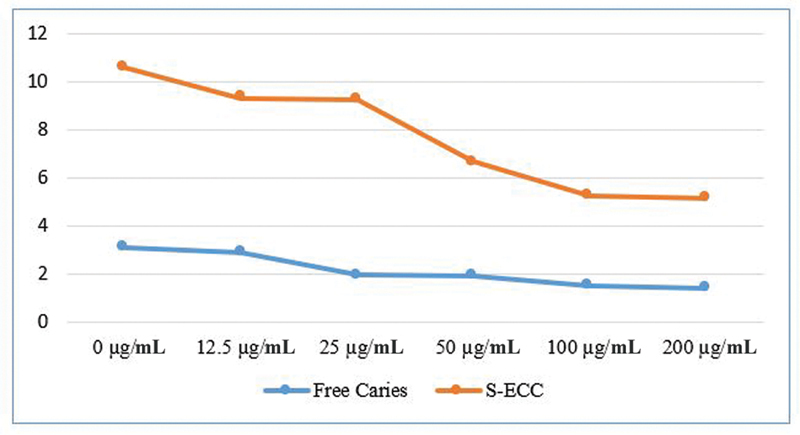
Graph of mean salivary neutrophil interleukin (IL)-1β expression after administration of various concentrations of *Centella asiatica* extract after 24 hours of incubation which was analyzed by flow cytometry and statistical *t* -test.

**Fig. 2:**
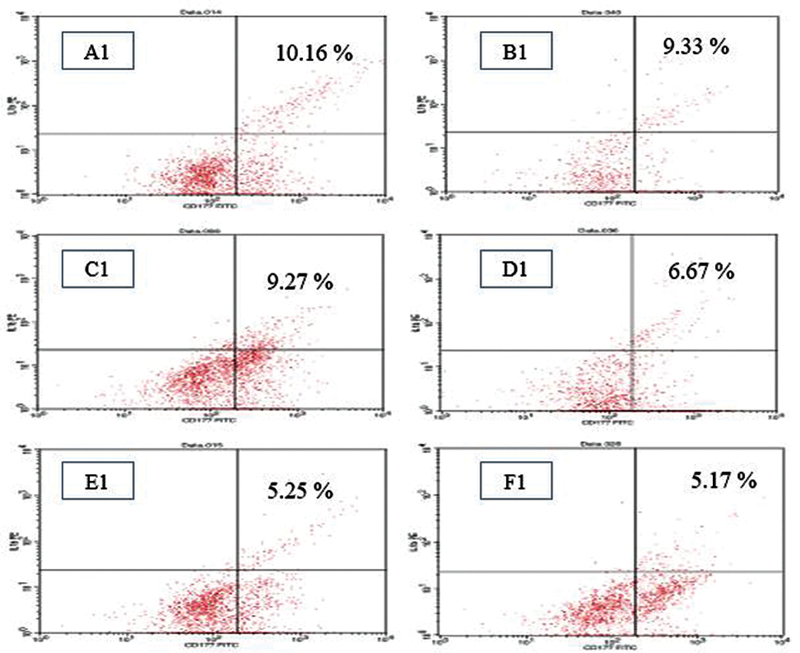
Salivary neutrophil interleukin (IL)-1 expression after administration of various concentrations of *Centella asiatica* (A1) extract. O g/mL (B1). 12.5 g/mL (C1). 25 g/mL (D1). 50 g/mL (E1). 100 g/mL (F1). 200 g/mL after 24 hours' incubation which was analyzed by flow cytometry test in caries-free severe early childhood caries (S-ECC) children.

**Fig. 3:**
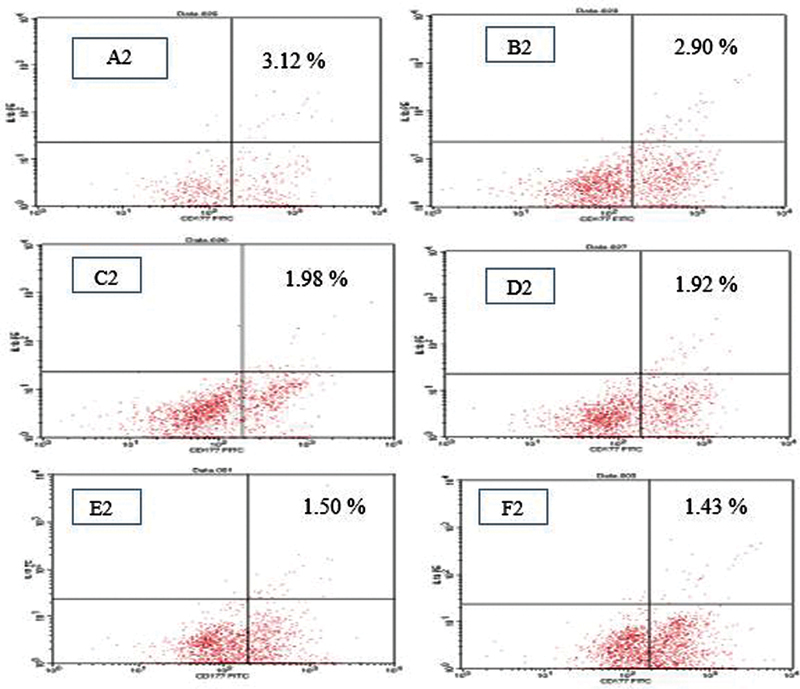
Salivary neutrophil interleukin (IL)-1β expression after administration of various concentrations of *Centella asiatica* extract (A2). 0 g/mL (B2). 12.5 g/mL (C2). 25 g/mL (D2). 50 g/mL (E2). 100 g/mL (F2). 200 g/mL after 24 hours' incubation which was analyzed by flow cytometry test in caries-free children.

The results of statistical analysis of the *t* -test in the caries-free group revealed that the administration of *C. asiatica* extract at a concentration of 12.5 g/mL resulted in a significant lowering in the expression of IL-1 on the surface of salivary neutrophil cells when compared with the control group. The larger the dose of *C. asiatica* , the lower the expression of IL-β ( Tables 1 and 2 ).

**Table 1:** The expression of IL 1-β on the surface of salivary neutrophils after administration of various concentrations of *Centella asiatica* extract after 24 hours in the free caries group

Group	*Centella asiatica* concentration (mg/mL)	Mean ± SD IL-1β level
Free caries	*Centella asiatica* 0%	3.12 ± 0.75
	*Centella asiatica* 12,5%	2.90 ± 0.57
	*Centella asiatica* 25%	1.98 ± 0.59
	*Centella asiatica* 50%	1.92 ± 0.66
	*Centella asiatica* 100%	1.50 ± 0.50
	*Centella asiatica* 200%	1.43 ± 0.51

Abbreviations: IL, interleukin; SD, standard deviation.

**Table 2:** Tukey HSD test for groups of *Centella asiatica* extracts of various concentrations (μg/mL) on the expression of IL-1β on the surface of salivary neutrophils in free caries

Concentration		*p* -Value of IL-1β expression on saliva neutrophil surfaces				
		Caries-free group (%)				
	0	12.5	25	50	100	200
0		0.977	0.006*	0.003*	0.000*	0.000*
12.5			0.043	0.026	0.000*	0.000*
25				1.000	0.609	0.462
50					0.731	0.588
100						1.000
200						

Abbreviations: HSD, honestly significant difference; IL, interleukin.

The results of the statistical analysis of the *t* -test in the S-ECC group revealed that the administration of *C. asiatica* extract at a concentration of 12.5 g/mL resulted in a significant decrease in the expression of IL-1 on the surface of salivary neutrophil cells when compared with the control group. The larger the dose of *C. asiatica* , the lower the expression of IL-β ( Tables 3 and 4 ).

**Table 3:** The expression of IL-1β on the surface of salivary neutrophils after administration of various concentrations of *Centella asiatic* *a* extract after 24 hours in the S-ECC group

Group	*Centella asiatica* concentration (mg/mL)	Mean ± SD IL-1β level	*p*
S-ECC	*Centella asiatica* 0%	10.6 ± 0.83	0.000*
	*Centella asiatica* 12,5%	9.33 ± 1.03	
	*Centella asiatica* 25%	9.27 ± 0.96	
	*Centella asiatica* 50%	6.67 ± 1.06	
	*Centella asiatica* 100%	5.25 ± 0.94	
	*Centella asiatica* 200%	5.17 ± 0.80	

Abbreviations: IL, interleukin; SD, standard deviation; S-ECC, severe early childhood caries.

**Table 4:** Tukey HSD test of the *Centella asiatica* extract of various concentrations (μg/mL) on the expression of IL-1β on the surface of salivary neutrophils in S-ECC

Concentration		*p* -Value of IL-1β expression on saliva neutrophil surfaces				
		S-ECC group (%)				
	0	12.5	25	50	100	200
0		0.500	0.422	0.000*	0.000*	0.000*
12.5			1.000	0.000*	0.000*	0.000*
25				0.000*	0.000*	0.000*
50					0.0141	0.030*
100						1.000
200						

Abbreviations: HSD, honestly significant difference; IL, interleukin; S-ECC, severe early childhood caries.

## Discussion

Inflammation is the immune system's response to harmful stimuli such as pathogens, damaged cells, poisonous substances, or irradiation. It works by removing harmful stimuli and initiating the healing process. Despite the fact that inflammation is necessary for the host's defense against these shocks, it must be carefully managed because excessive inflammation produces needless damage that leads to dysfunction and disease. The inflammatory response is first detected by the pathogen recognition receptor. 20 21

Intracellular signaling pathways are activated by inflammatory stimuli, which then activate the synthesis of inflammatory mediators. Primary inflammatory stimuli, such as microbial products and cytokines like IL-1β, IL-6, and tumor necrosis factor (TNF)-α, cause inflammation by interacting with Toll-like receptors (TLRs), IL-1 receptors, IL-6 receptors, and TNF receptors. 22

Tables 1 and 3 demonstrate that there was a decrease in IL-1 expression in both S-ECC and free caries after administration of *C. asiatica* extract. However, the expression was considerably higher in S-ECC than in free caries after administration of *C. asiatica* extract at the same dose. The immune system in children with S-ECC is more active as a result of exposure to *S. mutans* pathogens or microbial components, which are pathogen-associated molecular patterns (PAMPs). The quantity of *S. mutans* bacteria detected in S-ECC saliva cannot be acquired by adaptive immunity because T-cell receptor and its coreceptors, such as CD4, which can form complexes with class 2 major receptor histocompatibility complex receptors and antigens, do not function adequately. As a result, the number of *S. mutans* bacteria identified in the saliva of S-ECC children is larger than in the saliva of caries-free children. 23

Neutrophils, macrophages, and dendritic cells are the principal producers of IL-1β. On the other hand, gingival fibroblasts, periodontal ligament cells, and osteoblasts can all release IL-1β. 24 IL-1β secretion begins with the production of a proprotein, which is then proteolyzed into its active form by caspase-1. In response to PAMPs, such as lipopolysaccharide, or damage-associated molecular patterns (DAMPs), the inactive precursor, pro-IL-1, is created (DAMPs, e.g., HMGB1, ATP). PAMPs and DAMPs activate myeloid differentiation primary response signaling adapter 88 (MyD88) via pattern recognition receptors, namely TLRs. MyD88 activation causes IB breakdown, which is required for the release of nuclear factor kappa B (NF-κB) dimers and stimulates pro-IL-1 production. 15 High numbers of *S. mutans* were observed to cause numerous PAMPs to be identified by neutrophil cells in patients with S-ECC, resulting in increased IL-1β release. When microbial ligands are detected by suitable receptors, innate immunity effector cells release proinflammatory cytokines, which activate T and B cells, resulting in cell-mediated and humoral immune responses. 25 Activated monocyte macrophages create cytokines, which act as mediators of inflammation and immunological processes. This could be a problem for people who have S-ECC. The results showed that after receiving *C. asiatica* therapy, IL-1β levels reduced considerably. This suggests a robust link between the presence of tooth caries and the level of this inflammatory mediator. As a result, children with ECC are at risk of having high levels of stress. This cytokine has a higher concentration. 26 27

*C. asiatica* is a herbal plant that contains flavonoids, triterpene glycosides, vitamin C, or carotenoids which are high enough to have the potential to improve immune function. 28 According to Murray and Pizzorno, 29 pentacyclic triterpenoids, such as asiaticoside, brahmoside, and madecassic acid, as well as other components like centellose, centelloside, madecassoside, and quercetin, are all found in *C. asiatica* . The anti-inflammatory activity of quercetin can reduce the expression of the inflammatory genes TNF-α, IL-6, IL-1β, and cyclooxygenase-2, suppress nuclear activation factor (NF-κB), and limit the synthesis of c-Jun N-terminal kinase. 30 Several studies have investigated into the influence of quercetin on proinflammatory enzymes exposed with the dopaminergic neurotoxin 6-hydrooxydopamine, which causes nerve injury; quercetin reduces nitric oxide (NO) production and inducible NO synthase expression. 31

TLRs are one of the most important components of the immune system, as they play a key role in host cell identification and response to microbial infections. 19 TLRs are also known to activate the pathway, which regulates obesity-related inflammation and insulin resistance. Through upregulation of the TLR4 pathway, TLRs stimulate the release of proinflammatory cytokines by upregulating transcription factors such as NF-κβ and activated protein 1, resulting in increased proinflammatory reactions and polarization of M1 macrophages (classically activated macrophages). 32 33 *C. asiatica* , which contains quercetin, lowers inflammation by blocking the TLR4/NF-κβ signaling pathway.

## Conclusion

*C. asiatica* extract has the effect of reducing the proinflammatory cytokine IL-1β produced by salivary neutrophils on S-ECC via inhibiting NF-κB and mitogen–activated protein kinase signaling pathway activation and suggests that *C. asiatica* extract is a possible candidate for treating S-ECC.
